# Transcutaneous cervical vagus nerve stimulation (tcVNS) does not enhance learning and memory performance in a visual detection task

**DOI:** 10.1038/s41598-025-23365-5

**Published:** 2025-11-12

**Authors:** Dana Ravestein, Lucia F. Hendrikse, Gerard J. Veldhuis, Helena J. M. Pennings, Yvonne M. Fonken

**Affiliations:** 1 TNO, Netherlands Organization for Applied Scientific Research, Soesterberg, Netherlands; 2 Utrecht Center for Research and Development in Health Professions Education, University Medical Center Utrecht, Utrecht, Netherlands

**Keywords:** Transcutaneous cervical vagus nerve stimulation, Vagus nerve stimulation, Accelerate learning, Declarative memory, Response inhibition, Vigilance, Human behaviour, Cognitive neuroscience, Learning and memory, Consolidation, Hippocampus, Long-term memory, Short-term memory, Neuroscience, Psychology

## Abstract

Complex changes in our society demand continuous skill development for personal and professional readiness. Transcutaneous cervical vagus nerve stimulation (tcVNS) has the potential to enhance learning by non-invasively delivering electrical pulses to the cervical branch of the vagus nerve. This study aimed to replicate a previous study on the effect of tcVNS on learning. Thirty-four participants completed a four-day training in visual detection and recognition of specific vehicles in Synthetic Aperture Radar (SAR) images. Participants received either active or sham tcVNS before and after training. Performance was assessed immediately post-training and 1-, 30-, and 60-days after the last training. Contrary to expectations, active stimulation did not improve learning, vigilance, or response inhibition compared to sham stimulation. Exploratory analyses suggested a negative association between stimulation intensity and learning outcomes, consistent with an inverted-U dose–response relationship suggesting overstimulation. The absence of physiological biomarkers leaves unresolved whether stimulation failed to engage the vagus nerve, engaged it sub-optimally, or engaged it without downstream cognitive benefits. These findings underscore the importance of parameter optimization and biomarker validation in tcVNS research. Rather than a failed replication, this study offers a cautionary lesson for the field: even seemingly minor methodological choices may critically shape cognitive outcomes.

Trial registration. Registered 14/08/2023, registration number NL-OMON53198.

## Introduction

The rapid pace of technological advancements and evolving demands of the modern world require individuals to continuously acquire new skills and knowledge. This need for continuous learning is essential for maintaining effectiveness in changing professional roles and tasks. Moreover, extreme circumstances such as wartime or major public health events, can amplify the need for workers to swiftly acquire and enhance knowledge and skills. For example, during the COVID-19 pandemic there was an immediate need for more specialized intensive care unit workers and vaccination personnel, requiring large numbers of people to be trained in a very short amount of time^1^. In the military, fast-changing geopolitical developments can also create circumstances in which personnel need to be trained quickly and adequately. These examples highlight the societal importance of developing methods that can enhance the speed and durability of learning.

A promising new approach to boost learning and memory is transcutaneous vagus nerve stimulation (tVNS)^2,3^. tVNS is a non-invasive method of electrically stimulating the afferent fibers of the vagus nerve. Interest in tVNS as a cognitive enhancer originates from evidence with invasive vagus nerve stimulation (iVNS), which has been an approved treatment for refractory epilepsy since the 1990s^3–5^. Beyond its anti-epileptic effects, iVNS has shown to enhance cognitive functions, such as attention, decision-making, mood, and various forms of memory^3,4,6–8^. Importantly, several studies in epilepsy patients have demonstrated that iVNS administered during or shortly after learning can improve memory consolidation^6,8–10^. While these findings come from clinical populations, they provide a mechanistic foundation for extending investigation into healthy individuals.

The underlying rationale is rooted in neurobiology. Both invasive and non-invasive VNS are thought to indirectly activate the locus coeruleus-norepinephrine (LC-NE) system, a central neuromodulatory pathway that supports attention, learning, and memory^3,4,11^. The LC plays a crucial role in memory and learning by influencing multiple brain regions. For instance, it innervates the amygdala and hippocampus - structures essential for forming emotional and episodic memories^3,12^. It is also thought to send noradrenergic input to the thalamus and hypothalamus, which regulate sensory processing, wakefulness, and stress, as well as the prefrontal cortex, which governs executive functions like attention, response inhibition, and working memory^3^. By triggering NE release, VNS activates receptors that enhance neural plasticity and strengthen synaptic connections through long-term potentiation (LTP) - a key mechanism for memory formation. LTP boosts postsynaptic responses and improves synaptic transmission, both critical for consolidating new information^3,4,13^. Animal studies show that iVNS increases LTP in the hippocampus and promotes the expression of brain-derived neurotrophic factors (BDNF), immediate early genes (IEGs), and proteins essential for synaptic strengthening^3,13,14^. These molecular changes support long-term neural plasticity and memory consolidation^3^. In short, neural activation as response to VNS may support learning and memory via two primary mechanisms: (1) improving short-term alertness and attention, and (2) promoting neuroplasticity, and therefore, consolidation of the learned information.

The development of non-invasive ways to stimulate the vagus nerve has opened the door to studying the impact of non-invasive VNS on learning in healthy individuals. Two non-invasive methods, transcutaneous auricular vagus nerve stimulation (taVNS) and transcutaneous cervical vagus nerve stimulation (tcVNS), have shown promising effects on cognitive functioning. TaVNS targets the auricular branch of the vagus nerve at the outer ear, while tcVNS stimulates the vagus nerve in the neck. Both methods are hypothesized to engage neural pathways like those targeted by iVNS^15–17^. For instance, Frangos et al.^16,17^ demonstrated in an fMRI study that vagal projections can be accessed non-invasively via tcVNS, with observed brain activation patterns resembling those induced by iVNS and other forms of non-invasive vagus nerve stimulation such as taVNS. However, direct evidence of non-invasive VNS activating the vagus nerve and the locus coeruleus-norepinephrine (LC-NE) system in humans is lacking.

Although research on healthy participants is limited, existing studies suggest that both tcVNS and taVNS can enhance learning under certain conditions, though findings remain inconsistent. For instance, Jacobs et al.^18^ showed that applying taVNS during learning of name-face associations (i.e., encoding phase) and shortly afterwards (i.e., consolidation phase) improved recall of the learned associations in healthy participants. Similarly, Llanos et al.^19^ found that pairing taVNS with non-native Mandarin speech sounds enhanced speech category learning, particularly for the easier-to-learn tone categories. However, other studies have failed to find such benefits. Mertens et al.^20^ observed no significant memory improvements when taVNS was applied exclusively during consolidation, while Miyatsu et al.^21^ found no learning enhancement effects for taVNS but did observe positive effects for tcVNS.

While both taVNS and tcVNS are designed to stimulate the vagus nerve, their effects may diverge due to anatomical differences in stimulation sites. Some evidence suggests that tcVNS yields more consistent behavioral outcomes, particularly in clinical contexts^3^. Recent findings support tcVNS as a promising approach for enhancing learning and memory. For instance, Klaming et al.^22^ showed that tcVNS applied before a memory recognition task enhanced performance, likely by increasing attentional engagement during encoding. Likewise, McIntire et al.^2^ demonstrated that tcVNS delivered both before and after training improved learning outcomes, with benefits persisting 30- and 90-days post-training. These findings suggest that tcVNS may be especially effective for both short-term learning and long-term retention, particularly when stimulation is administered during both encoding and consolidation phases.

Nevertheless, these outcomes remain mixed, which may be due to variability in study designs and stimulation protocols. TcVNS can be administered with a wide range of stimulation parameters, including current intensity, pulse width, frequency, duty cycle, and session duration^23^. Further factors, such as the type of sham control, electrode placement, and side effects, can also influence outcomes. Yet the psychophysiological and cognitive effects of these parameters remain poorly understood, and there is currently no consensus on optimal protocols^23^. In addition, the absence of reliable biomarkers of vagal engagement further complicates interpretation or knowledge about the optimal protocol^32^. This uncertainty underscores the need for replication studies to evaluate the robustness of reported effects and to establish the boundary conditions under which tcVNS influences learning.

Given these uncertainties, the present study aimed to replicate and extend the findings of McIntire et al.^2^ whose study design we closely followed. The research question entailed whether tcVNS could enhance learning and support retention across days. Participants were trained to recognize specific vehicles in Synthetic Aperture Radar (SAR) images over four days; McIntire et al.^2^ used the same task. Based on the results of McIntire et al. [2], we hypothesized that participants who received tcVNS would show better performance on the SAR task during training (day 1–4), and increased retention of the learned task compared to participants in the “sham” condition (i.e., a placebo control condition in which the tcVNS device was placed in the neck instead of on the cervical branch of the vagus nerve and low intensity levels were applied without conductance gel). Moreover, we studied the effects of tcVNS on vigilance, cognitive control, and mental state, as McIntire et al.^2,22^ also found effects on some of these outcomes.

## Results

### SAR TASK

Of the thirty-four participants, two were removed from the analysis because their performance did not meet inclusion criteria (both were in the sham group). A total of thirty-two participants were included in the analysis, N_sham_ = 15 and N_active_ = 17. Out of thirty-two participants, thirty responded to the question asked after completion of the study regarding whether they believed they were in the active or sham group. In the sham group, 65% identified their group correctly, while 71% of participants in the active group guessed correctly.

On each of the sevendays, participants performed the SAR task consisting of two sub-tasks. First, they had to identify a specific target in radar images (target identification task); second, they had to detect changes in the image (change detection task) (for details on the tasks see section ‘Methods’). For each task, reaction time (RT) and accuracy were recorded, and higher accuracy as well as shorter reaction times indicate better performance. For the main analysis, the mean accuracy and mean RT for both the target identification and change detection tasks were calculated for each stimulation group and across days. Our main analysis involved comparing the learning performance (i.e., accuracy on SAR task) of the two groups (tcVNS vs. sham) over time. To do so, we performed a 2 (active tcVNS vs. sham; between-subjects) x 7 (number of testing days; within-subjects) repeated-measures MANOVA (rmMANOVA) on accuracy and RT of the two tasks (i.e., on 4 dependent variables).

**Fig. 1:**
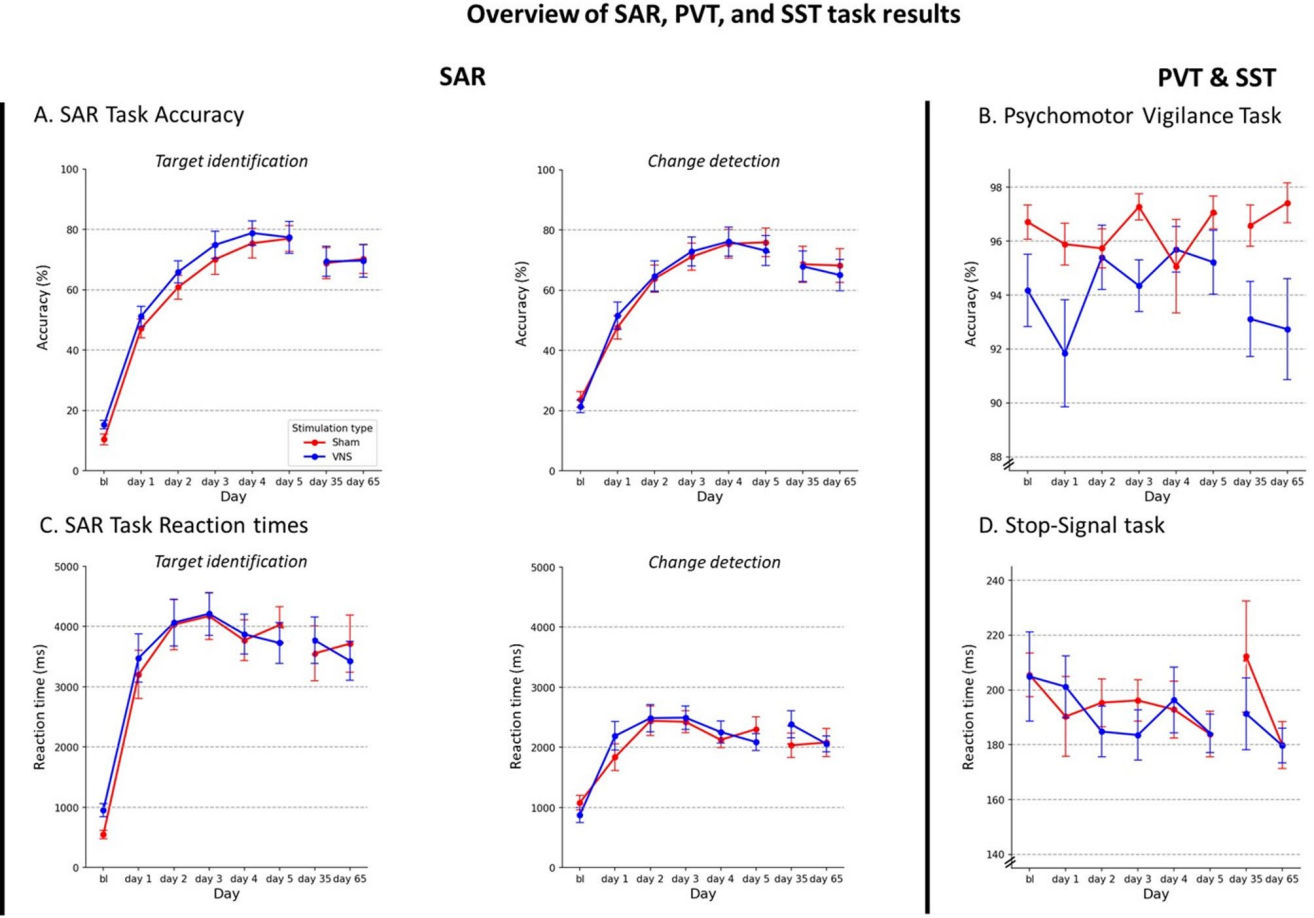
Overview of Synthetic Aperture Radar, Psychomotor Vigilance, and Stop-signal task results. Accuracy scores (panel **a**) and reaction times (panel **c**) for the two sub-tasks of the SAR task (target identification and change detection) across days for the active tcVNS group (blue) and sham group (red). Baseline measurements (bl) were performed on day 1 but excluded in the data analysis (see “Methods”). Panel **b** shows the accuracy score across days on the PVT. Panel **d** shows the SSRT across days on the SST. Error bars reflect standard error of the mean.

Figure 1 shows how accurate and fast participants were across days for the two stimulation groups, it shows the mean accuracy (panel **a**) and mean RT (panel **c**) on both subtasks (target identification, change detection) across days for the two stimulation groups (active tcVNS or sham). Accuracy on both subtasks increased over time, which indicates that learning took place. Statistical analysis (rmMANOVA) revealed a significant effect of day on accuracy on the SAR, *WTS*(24) = 78.75, *p* < .001. This increase in accuracy occurred both for target identification (*F*(3.46, 103.67) = 63.58, *p* < .001) and change detection (*F*(3.64, 109.26) = 45.24, *p* < .001), as follow-up analyses revealed. Post-hoc comparisons indicated that accuracy improved for both tasks only from day 1 until day 3 significantly, *ps* < .05, after which it plateaued. Additionally, reaction time across days increased significantly at both subtasks across days: target identification (*F*(3.07, 91.96) = 4.063, *p* = .009), change detection (*F*(3.54, 106.10) = 3.487, *p* = .043). However, this was for both subtasks only significantly the case between baseline and day 1, as the post-hoc comparison showed. Whether participants received active tcVNS or sham did not improve their performance more, since there was no main effect of stimulation on accuracy or reaction time, *WTS*(4) = 1.01, *p* = .91 or interaction effect between day and stimulation, *WTS*(24) = 20.27, *p* < .68.

In summary, these findings indicate a significant increase in accuracy on both subtasks, with no observed effect of stimulation.

### Psychomotor vigilance task (PVT)

Figure 1, panel **b** shows the accuracy scores for the PVT. For this task, data of four participants were excluded: three based on predetermined exclusion criteria for data variability and one due to corrupted data. Therefore, the analysis includes data from thirty participants. We wanted to compare vigilance scores across the two stimulation groups across days. To do so, we performed a 7 (days) x 2 (stimulation groups: active tcVNS vs. sham) factorial ANOVA, with the delta accuracy scores (accuracy score per day minus baseline accuracy score) as dependent variable. The analysis revealed no main effect of stimulation (*F*(1, 22) = 0.030, *p* = .864, no significant effect of day (*F*(3.34, 73.58) = 1.124, *p* = .348), nor an interaction effect (*F*(3.34, 73.58) = 2.00, *p* = .115).

### Stop-signal task (SST)

Figure 1, panel **d** shows the stop-signal reaction time (SSRT) for the SST. For the analysis of the SST data, one participant was excluded entirely due to predetermined exclusion criteria in data variability and 27 datapoints across all participants and days were excluded from the analysis due to race-model violations (i.e., when the mean RT on unsuccessful stop trials is numerically longer than the mean RT on go trials). For the main analysis, the mean SSRT was calculated for each stimulation group and across days. We wanted to compare the inhibition responses of the two stimulation groups over time. To do so, we performed a 2 (tcVNS vs. sham; between-subjects) x 7 (number of testing days; within-subjects) factorial ANOVA on SSRT. The analysis revealed no significant main effect of stimulation on SSRT, *F*(1, 26) = 0.78, *p* = .39. There was no significant effect of the day, *F*(3.57, 92.77) = 1.31, *p* = .27. The interaction between stimulation and day was also nonsignificant, *F*(3.57, 92.77) = 0.54, *p* = .69.

### Questionnaires

A 2 × 7 (same day and group factors as in the previous ANOVA’s) factorial ANOVA was performed on the positive and negative affect variables of the PANAS, to assess mood. The analysis revealed no effect of stimulation on positive or negative affect, all *F*’s < 0.182, and all *p*s > .67. Additionally, an ANOVA was conducted to compare the effects of stimulation and day on sleepiness, hours of sleep, and sleep quality. The ANOVA revealed significant effects of day on sleepiness, *F*(5.14, 149.05) = 5.28, *p* < .001, *η²* = 0.08, and hours of sleep, *F*(4.4, 127.47) = 3.16, *p* = .01, *η²* = 0.05, indicating variations across days. For sleep quality, there was a trend toward significance for day, *F*(4.27, 123.7) = 2.36, *p* = .053, *η²* = 0.04. No significant effects of stimulation or stimulation-by-day interactions were found for any sleep related variable (all *p*s > .17).

### Exploratory analysis stimulation intensity

**Fig. 2:**
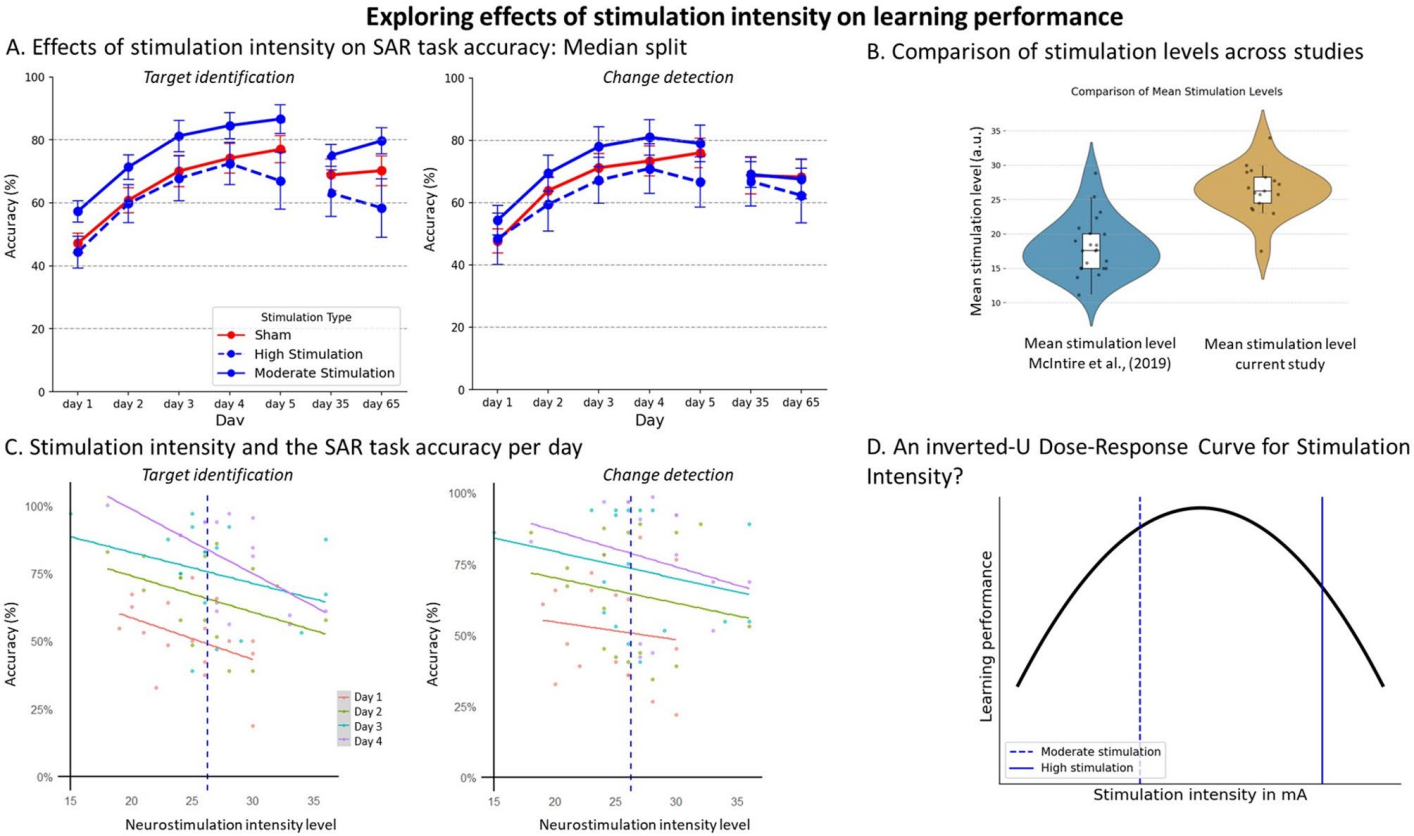
Exploratory analyses. Panel **a** Overview of accuracy scores separated by stimulation intensity for both sub-tasks. Data are the same as in Fig. 1**a**, except that the active stimulation group has been split into different stimulation intensity categories (moderate stimulation (< 26.25), and high stimulation (> 26.25) groups, sham = 10), based on a median split. Error bars reflect standard error of the mean. Note: median split has been conducted for visualization purposes only. Statistical analyses were done using stimulation intensity as continuous variable. Panel **b** Violin plot of the mean stimulation intensity across four days in both the McIntire et al., [2] and current study. Panel **c** Relationship between stimulation intensity and accuracy for both sub-tasks per day. The median is indicated with the vertical blue dotted line. Panel **d** The proposed inverted-U dose-response curve: moderate stimulation intensities are more optimal for improving learning performance compared to too low or too high stimulation.

We performed additional analyses to explore potential reasons why we did not find beneficial effects of tcVNS on learning, whereas McIntire et al.^2^ did. One difference we found was in tcVNS stimulation voltage (see Fig. 2**b**). In our study, the mean stimulation intensity over four days was 26.35 (*SD* = 3.47; scale 1–40 a.u.), which was significantly higher than the intensity reported by McIntire et al.^2^; *M* = 18.19, *SD* = 4.16), *t*(36.56) = 6.61, *p* < .001 (independent t-test).

To explore whether stimulation intensity might explain the null findings for stimulation effects reported above, we analyzed the relationship between stimulation intensity and task accuracy on the SAR task in the active stimulation group only. In Fig. 2**a**, for illustration only, we depict the eight participants with the highest tcVNS stimulation (i.e., those whose 4-day average stimulation level exceeds the median average of 26.25), moderate tcVNS (i.e., those whose average stimulation level is below the median average), and sham, separately. Visually, Fig. 2**a** suggests that participants who received high stimulation intensities, as well as those in the sham group, performed less accurately than participants who received moderate stimulation. This negative relationship between stimulation intensity and task performance is also evident on a day-to-day basis (Fig. 2**c**). These findings may reflect an inverted-U dose-response curve (Fig. 2**d**), where the stimulation intensities used in this study span the suboptimal range on the right side.

To confirm this impression, we conducted a linear mixed model analysis for both target identification and change detection accuracy separately, accounting for the nested structure within participants (e.g., each participant has multiple observations over days). In this analysis, only data from the active stimulation group were included, treating stimulation intensity as a continuous variable rather than the categorical grouping used for visualization. A linear mixed-effects model was fitted using the restricted maximum likelihood (REML) estimation method, with a random intercept per participant, a fixed intercept, and fixed effect of neurostimulation intensity and day. These exploratory analyses showed that neurostimulation level had a significant negative effect on target identification accuracy (*b* = -0.007, *p* = .018), indicating that higher stimulation intensity levels are associated with slightly reduced accuracy. No significant effect of neurostimulation level is found on change detection, *b* = 0.005, *p* = .362. In sum, these exploratory analyses provide an avenue for further research on the effect of stimulation and a possible inverted-U dose-response curve in learning.

To evaluate if stimulation intensity may have influenced accuracy on the PVT and SST, two separate mixed models analyses were performed (with the same 2 × 7 factors the previously mentioned mixed model). The stimulation intensity (continuous variable) and day showed no significant effect on accuracy of the PVT, (*F*(1, 48.44) = 2.01, *p* = .16) and (*F*(3, 38.29) = 0.79, *p* = .51) respectively. No significant effect of stimulation intensity (continuous variable) was found on the change in SSRT, *F*(1, 49.25) = 1.07, *p* = .31. Nor was an effect of day on change in SSRT found, *F*(1, 41.80) = 0.66, *p* = .60.

## Discussion

The present study examined the effects of transcutaneous cervical vagus nerve stimulation (tcVNS) on learning and cognition, aiming to replicate the positive outcomes reported by McIntire et al.^2^ and extend these findings by including a response inhibition task (SST) and assessments of mood and sleep. Participants received either active tcVNS or sham stimulation before and after training on a visual detection learning task (Synthetic Aperture Radar, SAR) across four consecutive days, with retention tests conducted at 1-, 30-, and 60-days post-training. Although participants improved on the learning task over time, tcVNS did not affect learning performance (i.e., accuracy on learning task), vigilance, response inhibition, sleep, or mood. These null findings contrast with prior studies showing positive tcVNS effects on these cognitive and behavioral outcomes^2,22,24,25^.

A primary potential explanation for the null findings of tcVNS on learning is the difference in stimulation intensity. In our study, stimulation was experimenter-controlled and increased until a visible lip pull was observed, whereas McIntire et al.^2^ employed a self-administered protocol that may have resulted in lower, and potentially more optimal, intensities. Participants may perceive lip pulls at lower intensities than can be visually detected by the experimenter. While our approach aimed to standardize stimulation, it may have inadvertently produced intensities above the optimal range for cognitive enhancement.

Our exploratory analyses supported this interpretation, revealing a small negative association between stimulation intensity and target identification accuracy, suggesting that high intensities may impair performance. This aligns with the inverted-U dose-response curve, which has been observed in both invasive and non-invasive VNS studies. For example, Helmsteadter et al.^10^ noted that memory improvements observed with low-intensity invasive VNS in epilepsy patients transitioned into performance suppression at higher intensities. Similarly, McHaney et al.^26^ reported better and faster learning with lower intensity taVNS in exploratory analyses. Hays et al.^27^ further highlighted that the relationship between VNS intensity and neuroplasticity follows an inverted U-pattern: low-intensity VNS does not sufficiently activate neural pathways, moderate-intensity VNS optimally enhances plasticity, while higher-intensity VNS hinders it. Animal studies also support this pattern. For instance, Borland et al.^28^ found that moderate stimulation intensities enhanced plasticity and cortical reorganization in the auditory cortex, whereas both lower and higher intensities failed to enhance plasticity (see also^29,30^). This inverted-U dose-response relationship may stem from nonlinear effects of norepinephrine release. Excessively high or low norepinephrine levels are associated with suboptimal neuromodulatory activation^27^, consistent with the Yerkes-Dodson law, which posits that an optimal level of arousal facilitates learning and performance^31^. Thus, it could be that stimulation intensity levels used in the present study may have exceeded the optimal range for cognitive enhancement, masking potential benefits of moderate levels. This could have led to performance deterioration in participants receiving higher stimulation levels, obscuring potential improvements in those exposed to moderate intensities. While these conclusions are tentative and based on exploratory analyses, they suggest that stimulation intensity could be a critical determinant of tcVNS efficacy and should be systematically manipulated in future studies.

A fundamental methodological limitation of our study is the reliance on lip pull as a proxy for vagus nerve engagement without physiological validation. While this approach was consistent with prior studies demonstrating tcVNS efficacy^2,22^, a lip pull reflects activation of adjacent motor nerves rather than afferent vagus nerve fibers. Additionally, tcVNS requires stimulation to pass through the skin in the neck and therefore currents are stronger and stimulation fields more diffused compared to other vagus nerve stimulation techniques^23^. This makes it possible that non-vagal cervical nerves or efferent fibers are also stimulated, making it harder to pinpoint the sole effect of stimulating the vagus nerve on cognition. Without physiological biomarkers, it is impossible to determine whether the null effects reflect ineffective vagal activation, excessively high intensity, or both. This represents a fundamental limitation: no definitive conclusions can be drawn about the mechanism of failure. Future studies must incorporate physiological biomarkers to assess vagal engagement, though non-invasive measures remain challenging and inconclusive^27,32,36^.

Several methodological constraints may have contributed to the null findings and should be considered when interpreting the results. First, the between-subjects design of the study may have introduced greater unexplained variance, reducing overall power and therefore complicating the detection of tcVNS effects, potentially masking subtle learning improvements. However, the study we attempted to replicate^2^ used a between-subjects design and still reported positive effects. Moreover, other work has found tcVNS effects on learning using between-subjects designs with heterogeneous samples and comparable sample sizes^24^. Second, blinding was not fully successful, as a substantial proportion of participants correctly guessed their assigned condition (71% active, 65% sham). This suggests that the sensation of the active device, or the absence of it in the sham condition, may have partially unblinded participants, highlighting the need for more credible sham protocols in future research. Third, differences in participant characteristics may have also contributed to null findings. Our sample was more heterogeneous than the military sample in McIntire et al.^2^, potentially increasing variability. Factors such as age, sex, and individual physiology (e.g., skin thickness, tissue conductivity, impedance) may influence tcVNS effectiveness^3^. Sex-specific differences may be particularly relevant: 47% of participants were female, compared to predominantly male samples in McIntire et al.^2^. Individualized dose-response relationships may have therefore masked group-level effects, underscoring the potential value of hierarchical modeling and adaptive stimulation protocols in future work. Fourth, task engagement may have further influenced the results. Participants in McIntire et al.^2^ may have been more familiar with and engaged in the SAR task due to their military background. In contrast, our participants’ lower engagement may have reduced tcVNS efficacy, as effective stimulation likely requires concurrent active learning^27^. Training accuracy remained low, averaging 46% on the fourth day, and was notably lower than SAR test scores. Additionally, the images used in the training were used multiple times and on the fourth day only 5% of the images were new, possibly contributing to lower interest and engagement. This reduced engagement may have limited tcVNS efficacy, which depends on concurrent active learning^27^. Nevertheless, overall performance pattern aligned with McIntire et al.^2^ indicating that task-related factors alone may not explain the absence of tcVNS effects. Taken together, these limitations underscore the need for future studies to adopt physiologically informed and individualized stimulation protocols, improve sham conditions, and account for participant heterogeneity in order to accurately assess the cognitive effects of tcVNS.

From a theoretical perspective, cognitive benefits of vagus nerve stimulation are thought to result from indirect stimulation of the locus coeruleus (LC), which increases the release of norepinephrine. This activation is believed to enhance short-term alertness and attention while promoting long-term neuroplasticity supporting memory consolidation. However, our results do not support these mechanisms behaviorally: tcVNS did not affect vigilance (measured by the PVT) or learning outcomes. It remains possible that tcVNS does not engage the LC in the same manner as invasive VNS or that our stimulation protocol failed to adequately activate the LC due to excessive intensity or individual differences in responsiveness.

While our study did not replicate the beneficial effects of tcVNS on learning reported by McIntire et al.^2^, it highlights critical factors - such as stimulation intensity, individual differences, and task engagement - that may modulate tcVNS efficacy. Importantly, this study highlights the need for physiologically informed and individualized stimulation protocols, careful sham design, and inclusion of objective biomarkers. Rather than a “failed” replication, these findings advance the field by identifying key methodological and mechanistic considerations necessary to optimize tcVNS for cognitive enhancement. Future research should address these factors, employ individualized stimulation protocols, and incorporate physiological measures to clarify if, and under what conditions, tcVNS can enhance learning.

## Methods

### Design and participants

This study employed a single-blind, placebo-controlled, between-subjects design with two groups (1:1 ratio). The experimental group received active tcVNS stimulation, while the control group received sham stimulation. We aimed to recruit 32 participants, as a power analysis (with G*Power^33^) indicated that this sample would provide an 80% chance to detect an effect of size = 0.25 in our repeated-measures MANOVA. Thirty-four participants (*M*_*age*_ = 30.5, female = 47%) took part in the study and were quasi-randomly assigned to either the active tcVNS (*N* = 17) or the sham condition (*N* = 17). The assignment was based on a randomly generated binary variable (0/1) in R, with additional adjustments to ensure an approximately equal distribution of age and gender across the two groups. One researcher made the allocation sequence, and two researchers enrolled participants before the test week started (i.e., data was sampled). We stopped the experiment since we had enough participants based on our power analysis, and due to time constraints. Participants had to be between 18 and 55 years old, in good health (e.g., free of viral infection symptoms in the five days preceding the study) and had to possess basic computer skills. All participants gave written and informed consent. Exclusion criteria included pregnancy, atherosclerosis, cardiovascular issues, epilepsy, psychiatric illness, the presence of active implanted metallic devices, concurrent use of other electronic devices, alcohol consumption the day before testing, and illicit drug use within the last three months. Attention disorders were added to the exclusion criteria after initial participant recruitment due to their potential confounding effects on performance in the tasks measuring attention. The study was conducted in a lab room at Science Park, Utrecht University. Recruitment took place from July 2023 to August 2024, and data was collected between August 2023 and September 2024. Participants received monetary compensation for their time, with a small completion bonus if all sessions were attended. The current study was evaluated and approved by the Medical Ethical Research Committee Brabant (P2317; NL84460.028.23) and conducted in accordance with the declaration of Helsinki. Two participants discontinued the study due to adverse events (vomiting and eye pain), likely unrelated to stimulation.

### Materials and instruments

#### Cervical transcutaneous vagus nerve stimulation

Cervical transcutaneous vagus nerve stimulation (tcVNS) was conducted using the gammaCore™ device, which is FDA- and EMA-approved for treating cluster headaches^34^. The device administers a small electrical current via two electrodes placed on the skin in the neck. The device featured 40 stimulation intensity levels, linearly corresponding to a maximum output of 60 milliAmps. Stimulation was applied twice per day, before and after the learning task. Each stimulation session consisted of two 2-minute stimulations with a 2-minute break in between, following standard protocol set by the manufacturer and used in previous studies showing the efficacy of tcVNS on cognition^2,22^. This resulted in a total of 8 min of stimulation per day. The electrical stimulation consisted of five sinusoidal (symmetrical biphasic) cycles of a 5000 Hz waveform at a rate of 25 Hz (duty cycle of 40 ms) with a peak voltage limited to 30 Volts, and a pulse width of 1000 µs^34^. The load impedance was 450–550 Ohms. Stimulation was applied by a trained researcher.

The number of stimulations and their duration were identical for both active and sham stimulation, but the stimulation procedure differed. For active stimulation, the device was placed on the right side in the neck over the cervical branch of the vagus nerve using conductive gel. The stimulation intensity was gradually increased until a visible lip pull response was elicited (*n* = 15) or the participant indicated that it felt too uncomfortable (*n* = 2). According to gammaCore™ instructions, a downward pull on the lip is indicative that the correct intensity level is reached^33^. Additionally, this procedure of increasing intensity until a lip pull was felt was based on previous studies demonstrating tcVNS efficacy on cognitive performance^2,22,23^. For sham stimulation, the device was placed on the posterior neck muscle (trapezius) without the use of conductive gel and set to a low stimulation intensity (maximum level = 10). This ensured that no actual stimulation was delivered. To maintain a double-blind design, an experimenter different to the one performing the stimulations, conducted the behavioral testing.

#### Tasks

**Fig. 3:**
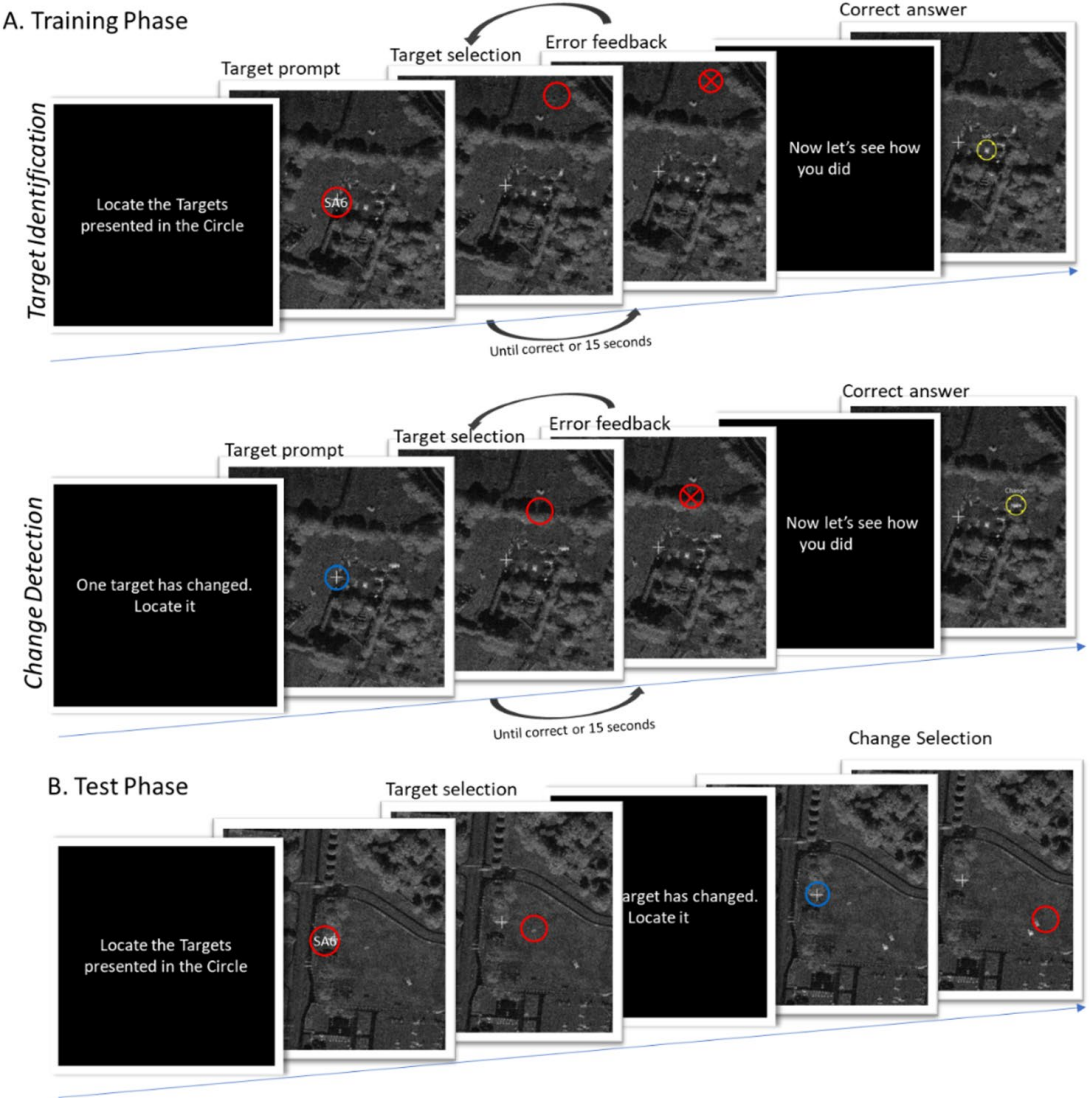
Schematic overview of the SAR task. In this task participants learned to recognize military vehicles in Synthetic Aperture Radar (SAR) images. Each trial involved the presentation of a SAR image with a prompt indicating which vehicle to locate. The participant subsequently clicks on the target location in the image (target identification). Next, the same SAR image is presented again with a small change, the participant clicks on the location of the change (change detection). **a**. During the training phase feedback is provided (a red "X" for wrong answers), the participant gets multiple attempts within 15 s before the correct location is shown with a yellow circle. **b.** During the testing phase no feedback is provided.

In order to replicate the effect reported by McIntire et al.^2^, we employed the same task using materials provided by the author. The Synthetic Aperture Radar (SAR) task is a visual detection task designed to assess target identification (identifying a target among distractors) and change detection (identifying changes in one image compared to a previous one) within radar images. The SAR task comprises two phases: a training phase lasting approximately 45 minutes, during which participants receive feedback on whether they responded correctly (to facilitate learning; see Fig. 3**a**), and a testing phase lasting about 25 minutes, during which no feedback is provided (see Fig. 3**b**). Participants all received instructions via an instruction video. The task has also been used in previous research, where it was shown that another form of stimulation (transcranial direct current stimulation) in combination with training led to better attained visual search accuracies^35^.

In each trial, participants were required to perform two consecutive tasks. First, they were presented with a radar image and were asked to locate and click on a specific vehicle within the image, completing the target identification task. Following this, the same image was displayed again with a slight modification, and participants were instructed to click on the location of the change, completing the change detection task. These changes could involve the addition, rotation, movement, or disappearance of a target vehicle. Across the training and testing phases, there were a total of 32 trials.

The primary outcome variables included reaction time (measured in milliseconds) and the percentage of correct responses, which are evaluated separately for target identification and change detection tasks. The task ran on Presentation^®^ software (Version 23.0, Neurobehavioral Systems, Inc., Berkeley, CA).

#### Other dependent variables

*Psychomotor Vigilance Task.* Participants completed a Psychomotor Vigilance Task (PVT), which is designed to measure the speed of responses to visual stimuli and assess vigilance^36^. In the PVT, participants are instructed to monitor a computer screen and press a response button as soon as a stimulus (red stopwatch) appears. The inter-stimulus interval (the time between the participant’s last response and the appearance of the next stimulus) is randomly sampled from 2 to 10 s. A valid response is followed by reaction time feedback. A response made before the stopwatch appears is an invalid response, which is followed by a brief error message. The main outcome measure is accuracy, which is calculated using the formula: Accuracy = Hits / (Hits + False Alarms + Lapses).

*Stop-signal task.* The stop-signal task (SST) is a variation of a go/no-go task designed to evaluate response inhibition, and action cancellation in particular^37^. An arrow pointing left or right serves as the go signal, instructing the participant to press the corresponding button (left/right). In one third of the trials a stop-signal in the form of an auditory tone is presented after a short delay (stop-signal delay), instructing the participant to inhibit their button-press. The delay between the arrow presentation and the signal beep can range from 50 milliseconds (minimum) to 1150 milliseconds (maximum), with adjustments made in 50-millisecond increments based on the participant’s performance (e.g., the task is made more difficult / the interval is lengthened, following a correct response). The initial Stop-Signal Delay is set to 250 milliseconds. There were 32 practice trials. There were three test blocks, each containing 64 trials, maintaining a 3-to-1 ratio of 48 no-signal and 16 signal trials in each block. The outcome measure is the stop-signal reaction time (SSRT), which is calculated from the go-RT distribution and the average stop-signal delay (see^38,39^. The SSRT is the time a person needs to successfully inhibit prepotent motor responses, with a smaller number indicating better inhibition and cognitive control of a participant^38–40^.

Both the PVT and SST task ran on the Millisecond software using Inquisit Lab^41,42^.

#### Questionnaires

To explore alternative reasons for why participants’ performance on the tasks differs, we also administered questionnaires every testing day measuring affect and sleep.

*Positive and Negative Affect Schedule.* The Positive and Negative Affect Schedule (PANAS;^43^ questionnaire is a brief scale comprised of 20 items. Ten items measure positive affect (e.g., alert, excited), and ten items measure negative affect (e.g., angry, afraid). Each item is rated on a five-point Likert Scale, where 1 = “Very Slightly or Not at All” and 5 = “Extremely”, indicating the extent of which a particular affect was experienced at that specific moment.

*Sleep questionnaire.* The Stanford Sleepiness Scale (SSS;^44^ measures sleepiness. Participants score how they feel on a 7-point scale ranging from ‘Wide awake’ (1) to ‘Falling asleep’ (7). Additionally, we asked participants about their total hours of sleep and the quality of sleep on a 5-point scale ranging from ‘bad’ to ‘very good’.

### Procedure

**Fig. 4:**
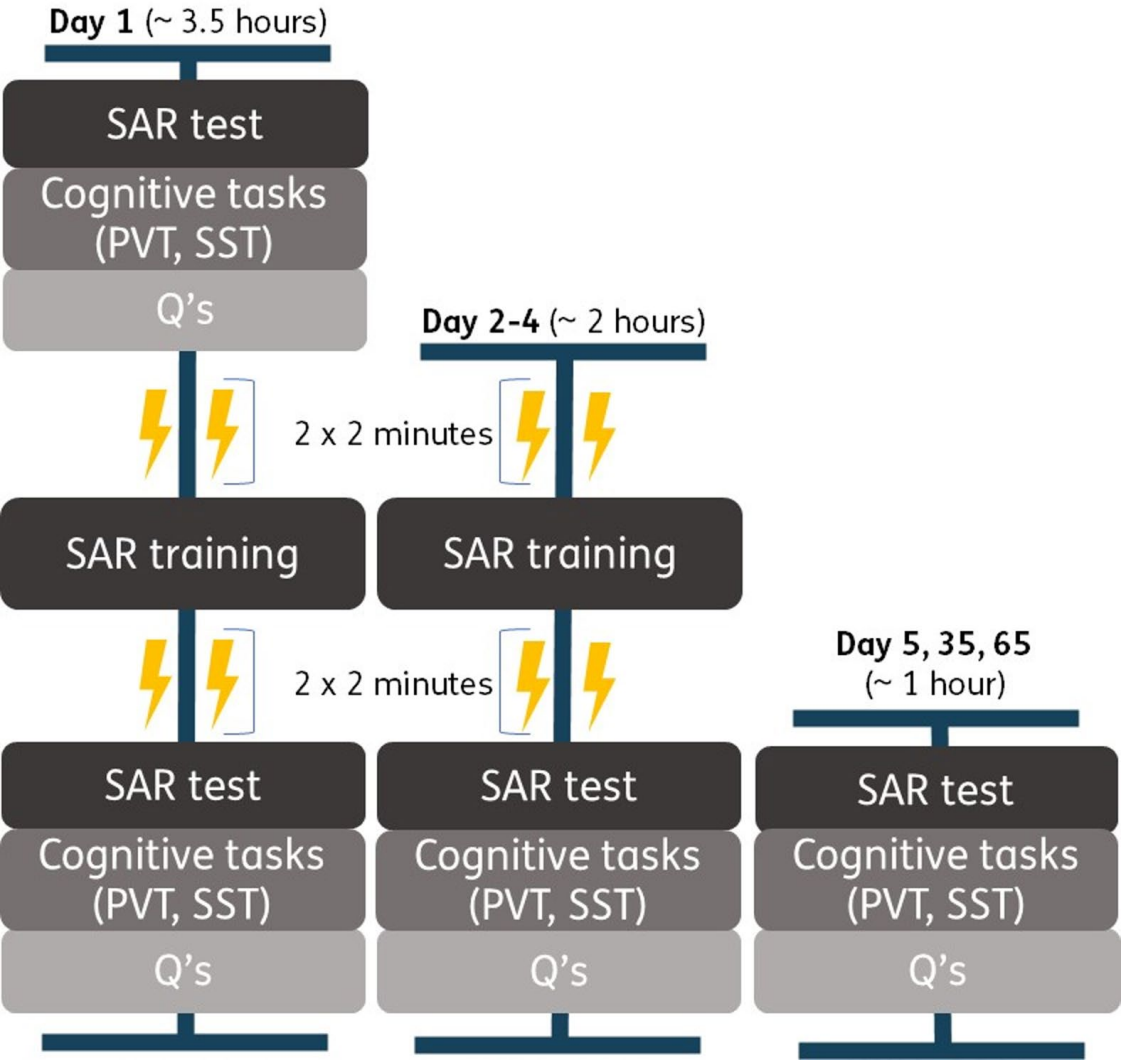
This figure visualizes the timeline of the study. The first day entailed the baseline measures (SAR, PVT, SST and questionnaires), stimulation, SAR training, stimulation again, and the tests (SAR, PVT, SST and questionnaires). The second to fourth day were without baseline and started directly with stimulation. The retention days (5th, 35th, and 65th day) consisted of the tests only.

Participants were recruited through our organization’s participant recruitment system, flyers at universities, and social media (i.e., LinkedIn, Facebook, and Instagram). After signing up through our recruitment system, participants were contacted for an intake phone call to ensure they understood the study and met all eligibility criteria. Over the course of the study, participants attended seven sessions at our research location. The sessions lasted between 1 (days 5, 35, and 65) and 3.5 h (day 1). Figure 4 provides a timeline of the data collection per session. The first five sessions occurred on consecutive days. On the first day, baseline measurements were taken. After the baseline assessment, participants received the SAR training, before and after which they received tcVNS or sham stimulation, and ended with the SAR test, PVT, SST, and the questionnaires.

Days 2 to 4 followed the same format but without the baseline measurements. On Day 5, 35, and 65 we tested whether training effects were retained, meaning participants only completed the SAR test, PVT, SST, and questionnaires.

### Data analyses

For the SAR test, participants’ performance on the fourth day was used to determine exclusion criteria. Participants whose average performance on either the target identification or change detection fell below two standard deviations (SDs) from the mean performance on day 4 were excluded from the analysis. This ensured that participants who showed no improvement or consistently underperformed were excluded.

On day 35, four participants were unable to attende. Their missing data were imputed using the group mean (active tcVNS or sham) for accuracy and reaction time (RT) on that specific day. To assess the effects of stimulation on learning, a multivariate repeated measures analysis of variance (rmMANOVA) was conducted, with time (7 days) as the within-subjects variable and stimulation group (active tcVNS vs. sham) as the between-subjects variable. The four dependent variables were RT and accuracy on both subtasks: target identification and change detection. Greenhouse-Geisser sphericity correction was applied to factors violating the sphericity assumption.

The baseline performance on the SAR task was not considered a true baseline, as participants received no prior instructions on the task. As such, the baseline likely reflects participants’ responses to uncertainty, rather than serving as a reliable measure of target identification or change detection. Consequently, the baseline was excluded from the analysis.

For the PVT and SST, participants were excluded if their performance fell below two standard deviations on more than three days. This criterion led to the exclusion of 4 participants (PVT) and 1 (SST) from the analysis. For the SST data, participants’ data were additionally excluded from specific days, if they violated assumptions from the race-model. A race-model violation occurs when the mean RT on unsuccessful stop trials is numerically larger than the mean RT on go trials, violating the independence assumption of the race model. This assumption posits an independent race between a go and a stop response. As recommended by Verbruggen et al.^38^, the SSRT estimates were not computed when this assumption was violated, as such violations make SSRT estimates unreliable. For the analysis of the cognitive tasks, PVT and SST, 7 (days) x 2 (stimulation groups: active tcVNS vs. sham stimulation) factorial analyses of variance (ANOVAs) were conducted.

Data were analyzed in R (v 2023.12.1) using the packages MANOVA.RM, rstatix, lme4, and lmerTest packages^45–48^. Data were visualized in Python using the Seaborn package^49^.

## Data Availability

The datasets generated during and/or analyzed during the current study are available from the corresponding author on reasonable request.
